# Parental Age and the Risk of ADHD in Offspring: A Systematic Review and Meta-Analysis

**DOI:** 10.3390/ijerph18094939

**Published:** 2021-05-06

**Authors:** Xianying Min, Chao Li, Yan Yan

**Affiliations:** Department of Epidemiology and Medical Statistics, Xiangya School of Public Health, Central South University, Changsha 410078, China; 196911004@csu.edu.cn (X.M.); JenniferLiChao@163.com (C.L.)

**Keywords:** ADHD, parental age, children, meta-analysis, attention deficit/hyperactivity disorder

## Abstract

Evidence has suggested that parental age at birth is a risk factor of offspring attention deficit/hyperactivity disorder (ADHD). We conducted a meta-analysis of observational studies investigating the association between parental age and offspring ADHD. We conducted a systematic search that followed the recommended guidelines for performing meta-analyses on PUBMED, EMBASE, and Web of Science up to 8 April 2021. We calculated pooled risk estimates from individual age with and without adjusting for possible confounding factors. Dose–response analysis for parental age and ADHD risk was performed. Eleven studies were selected in this meta-analysis, which included 111,101 cases and 4,417,148 participants. Compared with the reference points, the lowest parental age category was associated with an increased risk of ADHD in the offspring, with adjusted odds ratios (ORs) of 1.49 (95% confidence intervals (95%CI) 1.19–1.87) and 1.75 (95%CI 1.31–2.36) for the mother and father, respectively. The highest parental age was statistically insignificant, with adjusted ORs of 1.11 (95%CI 0.79–1.55) and 0.93 (95%CI 0.70–1.23) for mother and father separately. Dose–response analysis indicated a non-linear relationship of parental age with offspring ADHD, with the lowest ADHD risk at 31–35 years old. The results of this meta-analysis support an association between young parental age and the risk of ADHD. More high-quality studies are needed to establish whether the association with parental age is causal.

## 1. Introduction

Attention deficit/hyperactivity disorder (ADHD) is a persistent neurodevelopmental disorder of childhood and adolescence, with a worldwide epidemiological prevalence of 5.3% (5.01–5.56%). The core symptoms of ADHD are inattentiveness, impulsivity, and motor unrest [1,2]. ADHD symptoms affect social and learning ability and may last to adulthood [3]. Additionally, ADHD can impose a financial burden on society. The analysis revealed that estimates of ADHD-associated costs ranged from USD 831.38 to 20,538 per person, and from USD 356 million to 20.27 billion for every high-income country [4].

The etiology of ADHD is poorly understood. ADHD is a multifactorial disease, and both genetic and environmental risk factors can lead to child ADHD. Twin studies have revealed heritability estimates of about 70% [5]. However, gene–environment interactions exaggerated estimates of heritability [6]. In addition to heritability, 10–40% of the association with ADHD was accounted for by environmental factors [7]. Some perinatal risk factors such as maternal substance use and stress, parental age, and low birth weight are reported to contribute to child ADHD [8]. Recently, parental age at childbirth has been suggested as a possible risk factor for ADHD.

The fertility trends have changed in some countries. On the one hand, the average age of parents at childbirth has increased in high–income countries, and the proportion of women delivering in their thirties and forties has risen correspondingly [9,10,11]. On the other hand, adolescent childbearing still is a public health problem, and the rate of adolescent marriage and childbearing in the past decade rebounded [12]. Extensive evidence suggests that parental age at delivery is associated with outcomes in the offspring [13]. Advanced parental age may lead to a mental disorder such as autism spectrum disorder and schizophrenia [14,15]. Similarly, very young parents’ age increases rates of neurobehavioral outcomes in offspring, including behavioral disorders and intellectual disability [16].

A large number of studies have explored the association between parental age and ADHD in offspring. However, previous research regarding the association between parental age at childbirth and the rate of ADHD in offspring is inconsistent [17,18,19,20,21,22,23,24,25,26,27]. Although several studies have reported an insignificant association between ADHD in children and parental age at birth [17,26] most previous studies have reported younger age at birth as a risk factor of offspring ADHD [20,21,22,28]. Most studies support that lower parental age is a risk factor for offspring ADHD [16,18,20,21], but a few studies have found that advanced parental age increases risk for offspring ADHD [19,23]. It is essential to verify whether parental age is a risk factor of child ADHD, with the in-creasing trend of advanced parental age and adolescent childbirth. 

Establishing whether parental age at childbirth and ADHD in offspring are associated could have important public health implications. Although the pathogenesis of ADHD has not been clearly elucidated, the interaction between genes and environ-mental factors involves causal pathways [29]. The relationship between parental age and ADHD may provide clues to the etiologic factors leading to ADHD. Based on previous findings in autism and bipolar disorders regarding a dose–response association [30,31], we hypothesized finding a similar association in this analysis. Few meta-analyses have discussed the association between parental age and the risk of ADHD. Thus, this study aimed to systematically explore the association between pa-rental age at delivery and ADHD risk in offspring, and we also conducted a dose–response meta-analysis to quantitatively evaluate the association. The finding of a significant association would provide evidence for reducing the risk of offspring ADHD by intervening in reproductive age.

## 2. Methods

### 2.1. Systematic Literature Search

This study followed the guidelines of the Preferred Reporting Items for Systematic Reviews and Meta-Analysis (PRISMA) [32]. Two independent reviewers conducted a systematic literature search on PUBMED, EMBASE, and Web of Science up to 8 April 2021, for relevant studies which have been published. The search strategy was combined with the following keywords: (“*attention deficit disorder with hyperactivity*” OR “*ADHD*” OR “*attention–deficit/hyperactivity disorder*”) AND (“*maternal age*” OR “*paternal age*” OR “*parents age*”). The search was limited to studies published in the English language and conducted on human beings. Irrelevant studies were excluded by scanning the titles and abstracts. Additionally, referenced lists of the retrieved studies were checked manually for potentially relevant reports. 

### 2.2. Study Selection

The meta-analysis included observational studies examining the relationship between parental age at birth and the risk of ADHD in offspring. Studies were included if they: (1) measured the outcome in individuals aged 3–17 years; (2) examined maternal or paternal age as the variable; and (3) reported data on rates of ADHD stratified by at least two levels of parental age at childbirth or a summary estimate (i.e., relative risk, hazard ratio, or odds ratio) with its corresponding 95% confidence interval (CI). 

Studies were excluded from the final analysis if they were: (1) letters, reviews, conference abstracts, case reports, or protocols; (2) studies containing overlapping participants; or (3) studies without raw data. 

### 2.3. Data Extraction 

Two authors (Min and Li) independently reviewed the literature and extracted the information and data from the selected studies. To resolve discrepancies in the data collection process, the third author participated in the discussion. The corresponding authors were contacted for the full text and raw data that may not have been presented in the published articles. The following information and data were extracted from each article: author, year of publication, country, study design, sample size, number with ADHD, outcome, diagnostic assessment, and adjusted factors. If studies reported the risk estimates from several adjustment models, the estimates from the maximum adjustment ranges for potentially confounding variables were extracted.

Besides the number of each age group, the crude and adjusted effect size with corresponding 95% CI was extracted. ADHD was a rare outcome; therefore, the differences between RR, OR, and HR were ignored. OR was reserved to refer to all of them. In most included studies, the authors used the midpoint parental age category as the reference to calculate the odds ratio of ADHD based on the lower age categories versus the referenced group and the higher age categories versus the referenced group. Other studies only reported the risks of ADHD according to either higher age categories versus the lowest age category, or lower age categories versus the highest age category. 

### 2.4. Quality Assessment

As the Cochrane Collaboration recommended, the Newcastle–Ottawa Scale (NOS) [33] was used to assess the methodological quality of the included studies. The scale consists of 9 items. Studies scoring 0–3, 4–6, and 7–9 were regarded as low quality, moderate quality, and high quality, respectively. The two independent reviewers assessed the quality of each included study.

### 2.5. Data Synthesis and Statistical Analysis

Four pooled estimates of ADHD risk in children were evaluated from the meta-analysis: (1) the lower age categories vs. the referenced age categories of maternal age; (2) the lower age categories vs. the referenced age categories of paternal age; (3) the highest age categories vs. the referenced age categories of maternal age; and (4) the highest age categories vs. the referenced age categories of paternal age. Both crude and adjusted ORs were analyzed separately. The studies included in the analysis were conducted in different settings; therefore, I^2^ statistics and Q-statistics were used to explore the extent of heterogeneity among studies. If statistical heterogeneity was significant (I^2^ > 50% and *p* < 0.10), a random-effects model was used to estimate the overall OR and 95% CI. If considerable heterogeneity existed, subgroup analyses were stratified by study design, geographical area, and diagnostic method. Sensitivity analyses were conducted by omitting one study at a time. Publication bias was not performed because fewer than ten studies were included in the meta-analysis [34].

A two-stage random-effect meta-analysis was conducted to examine the dose–response relationship between maternal age and the risk of child ADHD. The lowest category was set as a reference group. When the average maternal age was not reported in the study, the midpoint of the upper and lower boundaries of each category was taken. When the lowest category and highest category were open intervals, the interval width was consistent with the adjacent one [35]. Generalized least squares (GLS) was used to construct linear and non-linear dose–response relationship models [36]. Non-linear dose–response meta-analysis of maternal age and the risk of child ADHD was performed using the restricted cubic splines with three knots (10%, 50% and 90%) [37]. The coefficient of the second regression spline was tested by chi-squared to judge whether there was a linear relationship. Regression parameters were assessed with a test level of α = 0.05. The dose–response relationships were shown graphically. All analyses were performed with STATA version 14.0 software (Stata Corp, College Station, TX, USA).

## 3. Result

### 3.1. Summary of Literature

The initial database search yielded 9078 records, with 7043 remaining after the removal of duplicates. Overall, 43 studies were retrieved for full-text assessment by screening titles and abstracts. Furthermore, 32 studies were excluded due to the reasons as follows: reviews or meta-analysis (*n* = 5), not related to exposure (*n* = 8), not associated with outcome (*n* = 9), not usable results (*n* = 10). 

Ten studies were removed from the meta-analysis mainly because they lacked data stratified by at least two levels of parental age. There were 4953 participants in ten excluded studies. Five studies were performed in Asia and Oceania [38,39,40,41,42]; three were conducted in America [43,44,45]; two were performed in Europe [46,47]. All of them only provided the average age of parents and calculated the effect size according to the average age. Even though the studies showed that parental age is associated with offspring ADHD [38,41,42,44,45,46,47], they were not included in this meta–analysis. 

**Figure 1:**
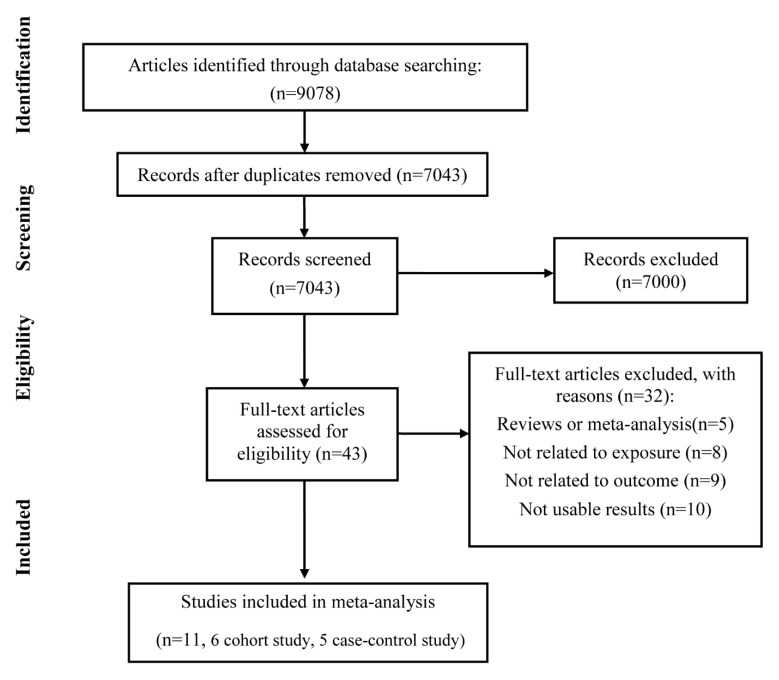
PRISMA flow diagram showing the detailed selection of eligible studies.

Finally, 11 studies, including six case-control studies and five cohort studies, were selected in this meta-analysis (Figure 1). There were 111,101 cases and 4,417,148 participants in total. Five studies were performed in Europe; three were conducted in North America; two were performed in Australia; and one was completed in Korea. According to the diagnosis of ADHD, six studies adopted *The Diagnostic and Statistical Manual of Mental Disorders, Fourth Edition (DSM–IV)*; meanwhile, four studies used *The International Classification of Diseases, 10th Revision (ICD–10)*. Each study adjusted for possible confounding factors. Nine studies adjusted for prenatal situation (Apgar score, birth weight, gestational age). Eight studies were adjusted for gender. Seven studies adjusted for substance exposure. Five studies adjusted for social–economic status. Only four studies adjusted for psychiatric history, and four studies adjusted for parental age. Characteristics of these included studies are summarized in Table 1. The detailed categories of parental age and odds ratios in each included study are listed in Table S1. According to the NOS, overall, the quality scores of the included studies were from 6 to 9, with an average score of 7.8 (Tables S2 and S3). 

### 3.2. Risk of Child ADHD According to the Lowest Parental Age Category vs. Reference Points

**Figure 2:**
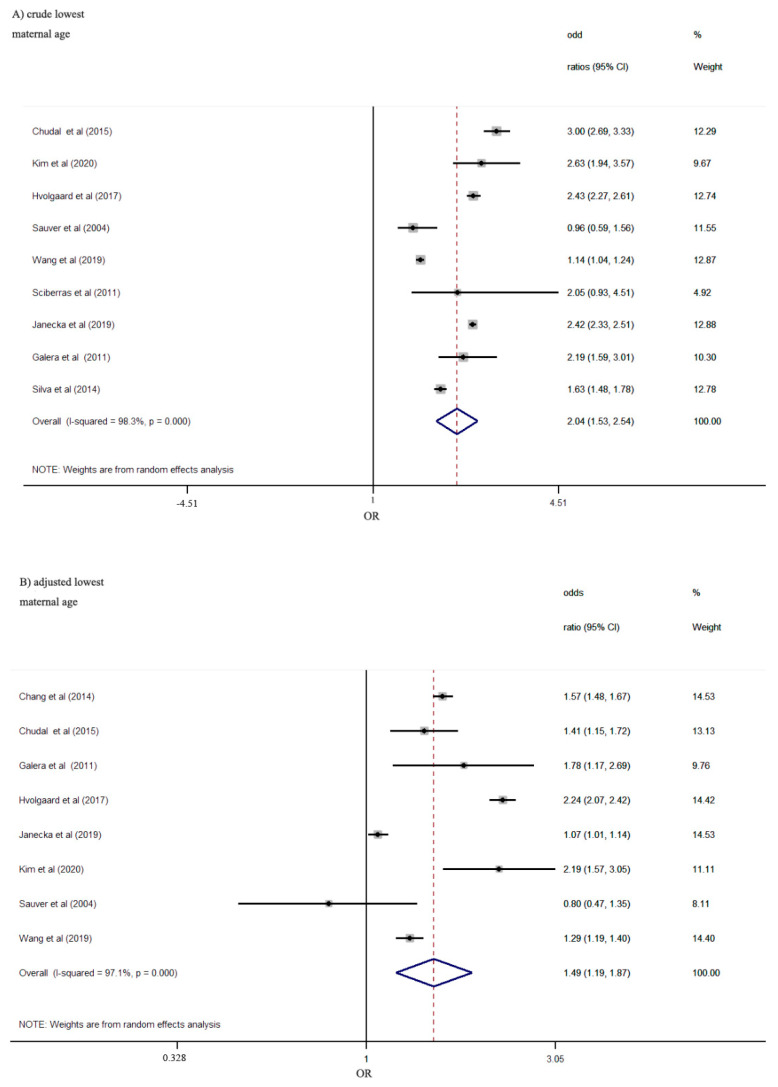
Risk of ADHD according to the lowest maternal age category vs. reference points. Pooled crude effects (**A**) and adjusted effects (**B**) for maternal age from random-effects meta-analyses are shown.

According to the pooled effect of crude ORs in nine studies, maternal lowest age at delivery was significantly associated with the increased risk of offspring ADHD (OR: 2.04; 95%CI 1.53–2.54). The association became weaker but persisted significantly after adjusting for potential confounding factors (OR: 1.49, 95%CI: 1.19–1.87) (Figure 2). Heterogeneity was significant for the crude estimate (I^2^ = 98.3%; *p* < 0.001) and the adjusted estimate (I^2^ = 97.1%; *p* < 0.001).

**Figure 3:**
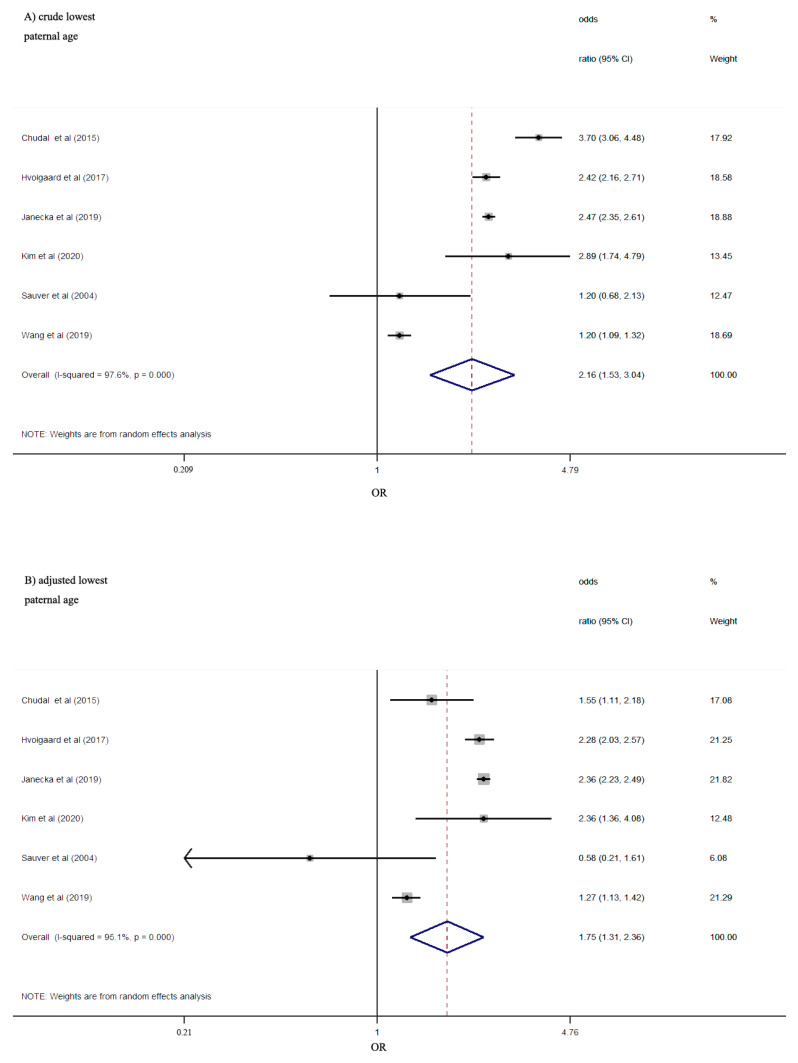
Risk of ADHD according to the lowest paternal age category vs. reference points. Pooled crude effects (**A**) and adjusted effects (**B**) for paternal age from random-effects meta-analyses are shown.

Six studies provided information about the lowest paternal age at delivery and the risk of ADHD in offspring. Similar to the results for maternal lowest age, the pooled estimates comparing lowest paternal age to reference age showed crude ORs of 2.16 (95%CI 1.53–3.04) and adjusted ORs of 1.75 (95%CI 1.31–2.36) (Figure 3), indicating an increased risk of ADHD in offspring. Statistically significant heterogeneity was found both in crude (I^2^ = 97.6%; *p* < 0.001) and adjusted (I^2^ = 95.1%; *p* < 0.001) estimate.

The association between young parents and risk of ADHD in offspring remained significant after adjusting for gender, prenatal condition, and substance exposure, with a 49% and 75% increased risk for offspring of mothers or fathers younger than 20 years, respectively. To explore heterogeneity, the results varied in some subgroup analyses stratified by study design, geographical area, and diagnostic method (Table 2). The results varied in some subgroup analyses. No statistically significant correlation between offspring ADHD and the lowest maternal age was found among studies conducted in America. Among case-control studies, studies in America, and studies with the DSM–IV diagnostic method, the adjusted estimated ORs showed no significant correlation between offspring ADHD and paternal lowest age. In the sensitivity analysis, the summary ORs did not change substantially by omitting one study in each turn, indicating that the results were reliable and stable.

### 3.3. Risk of Child ADHD According to the Highest Parental Age Category vs. Reference Points

The relationship between highest maternal age and ADHD in offspring was revealed in seven studies with crude ORs and five studies with adjusted ORs. The association was statistically non-significant either on crude ORs of 1.01(95%CI 0.89–1.16) or adjusted ORs of 1.11(95%CI 0.79–1.55) (Figure.S1). Heterogeneity was significant for the crude estimate (I^2^ = 71.3%; *p* = 0.002) and the adjusted estimate (I^2^ = 91.7%; *p* < 0.001).

Pooled effects of crude ORs suggested that no statistically significant correlation was observed between paternal highest age during pregnancy and the risk of offspring in five included studies (OR: 1.03, 95%CI: 0.85–1.24). After adjusting for confounding factors, the association was slightly changed but still non-significant (OR: 0.93, 95%CI: 0.70–1.23) (Figure S2). The heterogeneity was high for the crude (I^2^ = 91.9%; *p* < 0.001) and adjusted estimate (I^2^ = 94.7%; *p* < 0.001).

Subgroup analysis was conducted, and the results are presented in Table 2. No significant result was found, and the results were consistent in subgroup analyses. The results of sensitivity analysis indicated that pooled OR was not significantly changed by removing a single study per iteration. 

### 3.4. Dose–Response Meta-Analysis

**Figure 4:**
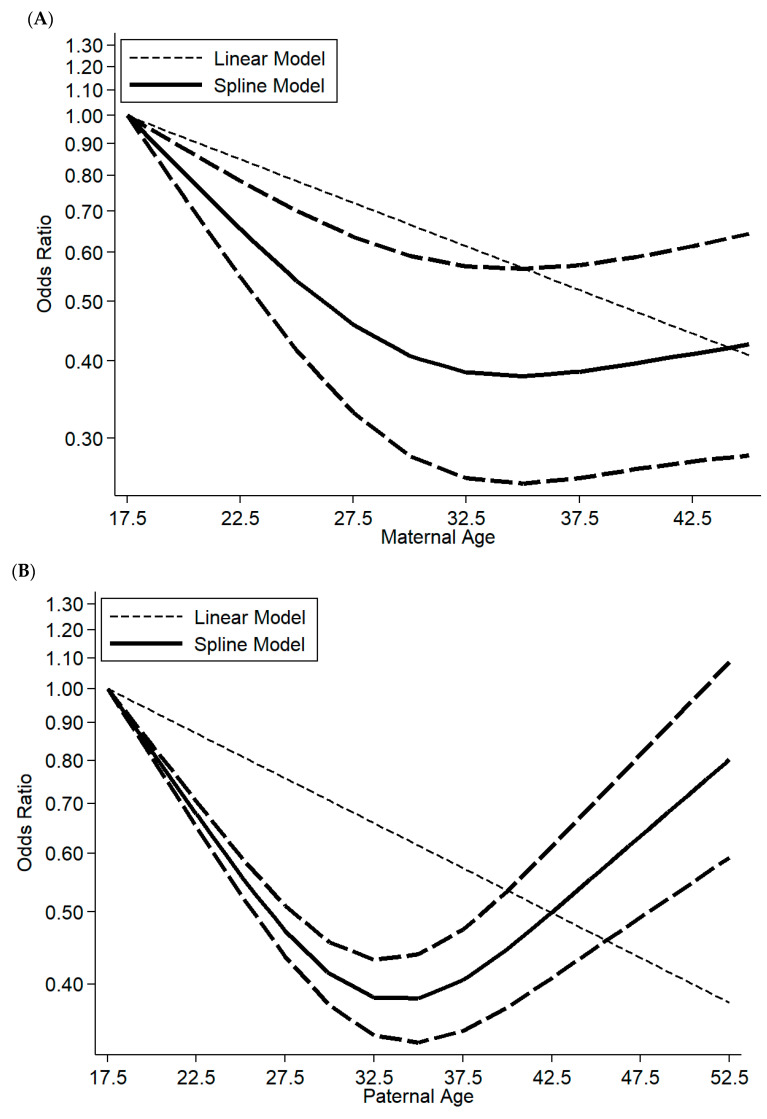
Dose–response association between (**A**) maternal age and offspring ADHD risk; (**B**) paternal age and offspring ADHD risk. Solid lines represent a relative risk; dashed lines represent 95% confidence intervals.

Five studies were suitable for dose–response meta-analysis on maternal age and the risk of offspring ADHD. Due to the results of heterogeneity (I^2^ = 98%, *p* = 0.04), a random-effects model was selected to merge the data. Non-linear relationships were found on maternal age and offspring ADHD, corresponding to *p* for nonlinearity (*p* = 0.032). The ORs for ADHD risk presented a U-shape. Compared with maternal age younger than 20 at pregnancy, the risk of offspring ADHD was the lowest when maternal age was 31–35 (OR: 0.48, 95%CI: 0.08–2.79). Children of mothers younger than 20 showed the highest OR for ADHD. However, a mother older than 40 did not significantly increase the risk of ADHD in her offspring. The dose–response relationship between maternal age and offspring ADHD is displayed in Figure 4A.

Similarly, the association between paternal age and the risk of offspring ADHD was non-linear (*p* = 0.007) in four studies eligible for dose–response meta-analysis. The results were similar to maternal age and are set out in Figure 4B. The lowest risk was found when fathers were 31–35 years old. A father who was younger than 20 or older than 45 was likely to increase the risk of ADHD in offspring in different degrees comparing with fathers at 31–35 years. Children of oldest fathers presented the second-highest rate for ADHD after children with younger fathers.

## 4. Discussion

The main results of the current meta-analysis were the following: (1) lower parental age was associated with an increased risk of ADHD in the offspring; (2) there was no significant association between advanced parental age and the increased risk of ADHD in the offspring; and (3) dose–response analysis revealed that parental age younger than 20 years had the highest risk of having an offspring with ADHD. The risk of ADHD in the offspring reduced as the parental age increased up to 31–35 years, while the risk of ADHD increased with the increase in parental age after 31–35 years old.

Meanwhile, there was high heterogeneity in this meta-analysis. Although subgroup analysis was conducted to detect the heterogeneity of the study, we are unlikely to explain heterogeneity adequately. Heterogeneity was decreased among the subset of geographic regions and diagnostic methods in the lowest parental age category. The sources of heterogeneity included the study design and diagnostic method in the highest maternal age category. In the paternal highest age category, the heterogeneity was reduced in the subset of study design. However, moderate heterogeneity still existed in subgroup analysis, and the heterogeneity of our study could not be totally attributed to study design, geographic regions, and diagnostic method. Additionally, the high heterogeneity may be related to the small number of studies included in the analysis. In the previous analysis between maternal age and offspring autism, the author suggested that the percentage of male offspring and year of diagnosis could be a source of heterogeneity [48]. We speculated that the sources of heterogeneity in our analysis may be similar to those in the previous analysis, because both ADHD and autism are neurodevelopmental disorders. Further studies are needed to explain the heterogeneity adequately.

When discussing the role of certain environmental factors in the development of ADHD, we must take into consideration that genetics and other environmental factors may be confounders. In our study, the adjusted ORs were consistent with the crude ORs, but both were lower than the crude ORs. The adjustment factors were different in each study, but most studies adjusted for child gender, prenatal condition, and substance exposure. This may indicate that these confounders have an impact on the final results, and these factors need to be controlled in future studies. ADHD is highly heritable; therefore, investigation of the association between parental age at childbirth and ADHD in offspring should consider parental psychiatric history [49]. In the study including sibling comparisons, the risk between parental age and offspring ADHD was attenuated but still existed [22]. Therefore, parental age might be a risk factor for offspring ADHD. In our study, the lowest risk was found when parents were 31–35 years old. This age is lower than the current average childbearing age [9]. In most studies included in this meta–analysis, 26–30 was set as a reference group, which may lead to bias in the risk of ADHD according to the lowest parental age category vs. reference category and highest parental age category vs. reference category.

In this study, parents younger than 20 years old had the highest risk of having offspring with ADHD. There are several plausible mechanisms by which parental age might affect ADHD in offspring. Some common characteristics of young parents may increase the risk of ADHD in offspring. Firstly, compared with children of older parents, those born to young parents are more often exposed to a disadvantageous family environment and parenting behavior, such as marital conflict, family disruption, parental smoking, and substance abuse [50,51,52,53]. Many types of research have shown that exposure to these adversities in early life subsequently leads to a risk of psychiatric disorders later in life [54,55]. Secondly, young mothers are more likely to have inadequate prenatal care and adverse birth outcomes such as low birth weight and prematurity [56,57]. Adverse birth outcomes are associated with ADHD in offspring [58,59]. Thirdly, young parents tend to have a history of hyperactivity problems, and the genetic susceptibility to ADHD in young parents may be transmitted to their offspring [60,61]. Moreover, the impulsivity associated with ADHD might lead to an increased rate of unplanned pregnancy, making young people become parents at an earlier age [62,63]. Thus, the risk of offspring ADHD with young parental age results from the interaction of genetic and environmental factors.

Advanced maternal age was not significantly associated with offspring ADHD, but offspring of fathers older than 45 years had an increased risk of ADHD in the dose–response analysis. In several previous studies, the results showed that advanced paternal age is a risk factor of autism, bipolar disorder, schizophrenia [14,64,65], but the association between advanced parental age and offspring ADHD is inconsistent. The increased occurrence of de novo mutations and epigenetic alternations with age increase is one possible explanation for the links between advanced paternal age and autism, BD, and schizophrenia [66,67,68,69]. Similar mechanisms may play a role in increasing ADHD risk in the offspring of older fathers. For older mothers, high reproductive age may lead to obstetric and perinatal complications, which have been proven to increase the risk of offspring ADHD [70,71]. However, advanced maternal age often means better maternal education and a more supportive home environment, which may improve children’s health and development [72]. There is no clear mechanism for the association between advanced parental age and ADHD symptoms in offspring. Further studies should focus on the explicit mechanism of how parental age at childbirth causes the risk of ADHD in offspring.

To the best our knowledge, this is the first meta-analysis providing comprehensive insights into the effects of parental age on offspring risk of ADHD based on current observational studies. Additionally, all the ADHD diagnoses were based on the clinical record in the included studies. However, we should be cautious in explaining the results with several limitations of this study. Firstly, some important confounders were not fully adjusted in the included studies, which may affect the accuracy of the conclusions. Generally, maternal age was related to paternal age, and vice versa. However, some studies did not take the other parent’s age into account as a confounding factor. It seems necessary to adjust the effects between the two effects and note the potential collinearity. Secondly, the small number of studies and sample size in some subgroups reduced the statistical power and led to inaccuracy in some of the pooled risk estimates. Thirdly, in most of the included studies, the authors used a midpoint of parental age categories as the reference group. We could only assess the risk of ADHD based on the lowest or highest parental age category versus the reference group. According to the current literature, the risk of ADHD based on the highest vs. lowest parental age was unable to be assessed. It seems desirable that future studies should proceed to meticulous analyses with appropriate categorical approaches. Moreover, extreme age subgroups should be considered if possible.

## 5. Conclusions

This meta-analysis showed the link between parental age and the risk of offspring ADHD. However, the association should be interpreted with caution because the available evidence consists of observational studies and it may be confounded by potential factors. Recognition of this risk is of great importance in both clinical psychiatric practice and as a public health issue. Parental age is one of the preventable risk factors of adverse outcomes in the offspring. Future public health policies should pay more attention to the children of old parents or young parents and provide appropriate parenting guidance. Further studies are needed to explicitly demonstrate the casual relationship and mechanisms between parental age and risk of ADHD in the offspring.

## Figures and Tables

**Table 1:** Characteristics of included studies.

Study	Study Site	Study Design	*n*	Number with ADHD in Study	Outcome	Diagnostic Method	Type of Adjusted Factors ^a^					
							Gender	SES	Psyc. History	Prenatal	Parental Age	Exposure
Chang et al. (2014) [23]	Sweden	Cohort	1,495,543	30,674	ADHD	ICD–10	✓			✓	✓	
Chudal R. et al. (2015) [20]	Finland	Case-control	49,534	10,409	ADHD	ICD–10		✓	✓	✓	✓	✓
Galera et al. (2011) [25]	Canada	cohort	2057	330	ADHD	DSM–IV	✓	✓	✓	✓		✓
Gustafsson et al. (2011) [18]	Sweden	Case-control	32,012	237	ADHD	DSM–III–R, DSM–IV	✓			✓		✓
Hvolgaard et al. (2017) [22]	Danish	Sibling cohort	943,785	12,294	ADHD	ICD–10	✓			✓	✓	✓
Janecka et al. (2019) [21]	Danish	Cohort	1,490,745	25,307	ADHD	ICD–10	✓		✓	✓		✓
Kim et al. (2020) [19]	Korea	Case-control	28,973	2112	ADHD	K–ARS	✓	✓		✓		✓
Sauver et al. (2004) [26]	America	Case-control	5701	305	ADHD	DSM–IV	✓	✓		✓		
Sciberras et al. (2011) [17]	Australia	Cohort	4464	57	ADHD	DSM–IV						
Silva et al. (2014) [24]	Australia	Case-control	43,062	12,991	ADHD	DSM–IV						
Wang et al. (2019) [27]	America	Cohort	321,272	16,385	ADHD	DSM–IV	✓	✓	✓	✓	✓	✓

Note: SES, social–economic status. ^a^ Confounding represents model covariates for gender, social–economic status (parental educational level, household income), psychiatric history (parental psychiatric history), prenatal situation (birth order, birth year, birth weight, gestational age, Apgar score, history of comorbidity), parental age (adjusted maternal age for paternal age), exposure (prenatal tobacco exposure, prenatal alcohol exposure, prenatal illegal drug exposure).

**Table 2:** Subgroup analyses for studies included in the analysis.

	Maternal			Paternal		
Subgroup Analysis	*n*	OR (95% CI)	Heterogeneity (I^2^, *p*)	*n*	OR (95% CI)	Heterogeneity (I^2^, *p*)
Lowest vs. referred (crude)						
Design						
Case-control	4	1.94 (1.26, 2.99)	I^2^ = 96.4%, *p* < 0.001	3	2.44 (1.30, 4.57)	I^2^ = 85.4%, *p* = 0.001
Cohort	5	1.96 (1.41, 2.73)	I^2^ = 98.4%, *p* < 0.001	3	1.93 (1.22, 3.05)	I^2^ = 98.8%, *p* < 0.001
Geographical area						
Europe	3	2.57 (2.32, 2.85)	I^2^ = 85.8%, *p* = 0.001	3	2.74 (2.28, 3.29)	I^2^ = 88.0%, *p* < 0.001
America	3	1.35 (0.85, 2.14)	I^2^ = 87.3%, *p* < 0.001	2	1.20 (1.09, 1.32)	I^2^ = 0%, *p* = 0.97
Asia and Oceania	3	2.02 (1.38, 2.96)	I^2^ = 77.5%, *p* = 0.012	1	2.89 (1.74, 4.80)	
Diagnostic method						
ICD–10	4	2.58 (2.34, 2.83)	I^2^ = 79.0%, *p* = 0.003	4	2.75 (2.32, 3.26)	I^2^ = 82.3%, *p* = 0.001
DSM–IV	5	1.48 (1.12, 1.94)	I^2^ = 90.4%, *p* < 0.001	2	1.20 (1.09, 1.32)	I^2^ = 0%, *p* = 0.97
Lowest vs. referred (adjusted)						
Design						
Case-control	5	1.52 (1.15, 2.01)	I^2^ = 98.3%, *p* < 0.001	3	1.48 (0.84, 2.62)	I^2^ = 65.3%, *p* = 0.056
Cohort	3	1.41 (0.90, 2.21)	I^2^ = 81.5%, *p* = 0.005	3	1.90 (1.31, 2.75)	I^2^ = 97.9%, *p* < 0.001
Geographical area						
Europe	4	1.52 (1.08, 2.13)	I^2^ = 98.6%, *p* < 0.001	3	2.22 (1.96, 2.52)	I^2^ = 66.3%, *p* = 0.051
America	3	1.27 (0.93, 1.75)	I^2^ = 63.6%, *p* = 0.066	2	1.02 (0.51, 2.03)	I^2^ = 55.5%, *p* = 0.134
Asia and Oceania	1	2.19 (1.57, 3.05)		1	0.36 (1.36, 4.09)	
Diagnostic method						
ICD–10	5	1.62 (1.19, 2.20)	I^2^ = 98.2%, *p* < 0.001	4	2.24 (2.01, 2.51)	I^2^ = 49.5%, *p* = 0.115
DSM–IV	3	1.27 (0.93, 1.75)	I^2^ = 63.3%, *p* = 0.066	2	1.02 (0.51, 2.03)	I^2^ = 55.5%, *p* = 0.134
Highest vs. referred (crude)						
Design						
Case-control	4	1.08 (0.90, 1.30)	I^2^ = 57.8%, *p* = 0.068	3	1.20 (0.89, 1.63)	I^2^ = 76.3%, *p* = 0.015
Cohort	3	0.94 (0.77, 1.15)	I^2^ = 69.7%, *p* = 0.037	2	0.87 (0.71, 1.07)	I^2^ = 95.2%, *p* < 0.001
Geographical area						
Europe	3	0.95 (0.81, 1.13)	I^2^ = 77.4%, *p* = 0.012	3	0.90 (0.75, 1.07)	I^2^ = 90.9%, *p* < 0.001
America	1	1.11 (0.80, 1.54)		1	1.07 (0.84, 1.36)	
Asia and Oceania	3	1.13 (0.82, 1.56)	I^2^ = 71.0%, *p* = 0.032	1	1.65 (1.27, 2.15)	
Diagnostic method						
ICD–10	4	1.05 (0.85, 1.30)	I^2^ = 83.6%, *p* < 0.001	4	1.02 (0.83, 1.26)	I^2^ = 93.5%, *p* < 0.001
DSM–IV	3	1.00 (0.87, 1.14)	I^2^ = 0%, *p* = 0.726	1	1.07 (0.84, 1.36)	
Highest vs. referred (adjusted)						
Design						
Case-control	3	1.10 (0.72, 1.67)	I^2^ = 78.2%, *p* = 0.010	3	1.20 (0.95, 1.51)	I^2^ = 0%, *p* = 0.548
Cohort	2	1.12 (0.54, 2.30)	I^2^ = 97.4%, *p* < 0.001	2	0.73 (0.51, 1.05)	I^2^ = 98.3%, *p* < 0.001
Geographical area						
Europe	3	0.99 (0.65, 1.51)	I^2^ = 94.8%, *p* < 0.001	3	0.81 (0.59, 1.10)	I^2^ = 96.7%, *p* < 0.001
America	1	1.11 (0.80, 1.54)		1	1.99 (0.70, 5.64)	
Asia and Oceania	1	1.82 (1.05, 3.16)		1	1.22 (0.90, 1.65)	
Diagnostic method						
ICD–10	4	1.11 (0.75, 1.65)	I^2^ = 93.4%, *p* < 0.001	4	0.88 (0.66, 1.18)	I^2^ = 95.8%, *p* < 0.001
DSM–IV	1	1.11 (0.80, 1.54)		1	1.99 (0.70, 5.64)	

## Supplementary Materials

The supplementary materials are available online at https://www.mdpi.com/article/10.3390/ijerph18094939/s1, Table S1. Categories of parental age group and odds ratios of ADHD in the included studies; Table S2. Quality assessment of the included studies (case-control studies); Table S3. Quality assessment of the included studies (cohort studies); Figure S1. Risk of ADHD according to the highest maternal age category vs. reference points; Figure S2. Risk of ADHD according to the highest paternal age category vs. reference points.
