# Supplementary material for: Parental Age and the Risk of ADHD in Offspring: A Systematic Review and Meta-Analysis
Source: Int J Environ Res Public Health. 2021 May 6;18(9):4939. doi: 10.3390/ijerph18094939 (PMC8124990; doi:10.3390/ijerph18094939)
Supplement: Supplementary file 1 [file ijerph-18-04939-s001.zip › ijerph-1178483-supplementary.pdf]

Table S1. Categories of parental age group and odd ratios of ADHD in the included studies

Study	Mother or father	Categories of parental age group(years)	Crude or Adjusted	Case	Number	OR (95% CI)
Chang et al, 2014	Maternal	≤20	Adjusted	NA		1.57(1.48,1.67)
		＞20	Adjusted			1
Chudal, R et al, 2015	Maternal	<20	Crude	696	1647	3.00(2.69,3.33)
		20-24	Crude	2459	8710	1.59(1.50,1.69)
		25-29	Crude	3411	17094	1
		30-34	Crude	2479	14437	0.83(0.78,0.88)
		35-39	Crude	1099	6291	0.85(0.79,0.91)
		≥40	Crude	265	1354	0.98(0.85,1.13)
	Maternal	<20	Adjusted	696	1647	1.41(1.15,1.72)
		20-24	Adjusted	2459	8710	1.22(1.11,1.34)
		25-29	Adjusted	3411	17094	1
		30-34	Adjusted	2479	14437	0.87(0.80,0.94)
		35-39	Adjusted	1099	6291	0.85(0.75,0.95)
		40-44	Adjusted	265	1354	0.79(0.64,0.97)
	Paternal	<20	Crude	221	449	3.70(3.06,4.48)
		20-24	Crude	1492	5033	1.63(1.51,1.75)
		25-29	Crude	2951	14151	1
		30-34	Crude	2870	15774	0.84(0.80,0.89)
		35-39	Crude	1607	8748	0.85(0.79,0.91)
		40-44	Crude	627	3268	0.89(0.81,0.98)
		45-49	Crude	231	1034	1.10(0.94,1.28)
		≥50	Crude	70	336	0.99(0.75,1.29)
	Paternal	<20	Adjusted	221	449	1.55(1.11,2.18)
		20-24	Adjusted	1492	5033	1.20(1.07,1.34)
		25-29	Adjusted	2951	14151	1

		30-34[re]	Adjusted	2870	15774	1.03(0.95,1.12)
		35-39	Adjusted	1607	8748	1.07(0.97,1.19)
		40-44	Adjusted	627	3268	1.09(0.94,1.26)
		45-49	Adjusted	231	1034	1.26(1.009,1.58)
		≥50	Adjusted	70	336	1.08(0.73,1.58)
Galera et al, 2011	Maternal	＜21	Crude	NA		2.19 (1.59–3.01)
		⩾21	Crude			1
		＜21	Adjusted	NA		1.78 (1.17–2.69)
		⩾21	Adjusted			1
Gustafsson et al, 2011	Maternal	5 year increase	Adjusted			0.86(0.76,0.98)
Hvolgaard et al, 2017	Maternal	≤20	Crude	990	31339	2.43 (2.27,2.61)
		21-25	Crude	2284	114086	1.54 (1.47,1.61)
		26-30	Crude	6882	529385	1
		31-35	Crude	1243	115199	0.83 (0.79,0.88)
		＞35	Crude	895	81960	0.84 (0.78,0.90)
	Maternal	≤20	Adjusted	NA		2.24 (2.07,2.42)
		21-25	Adjusted			1.49 (1.42,1.56)
		26-30	Adjusted			1
		31-35	Adjusted			0.80 (0.76,0.85)
		＞35	Adjusted			0.78 (0.72,0.84)
	Paternal	≤20	Crude	310	9854	2.42 (2.16,2.71)
		21-25	Crude	1450	73381	1.52 (1.44,1.60)
		26-30	Crude	7212	554770	1
		31-35	Crude	1743	161539	0.83 (0.79,0.87)
		＞35	Crude	1579	153749	0.79 (0.75,0.83)
	Paternal	≤20	Adjusted	NA		2.28 (2.03,2.57)
		21-25	Adjusted			1.49 (1.40,1.57)
		26-30	Adjusted			1
		31-35	Adjusted			0.78 (0.74,0.82)
		＞35	Adjusted			0.61 (0.57,0.65)

Janecka et al, 2019	Maternal	<22.5	Crude	3020	63027	2.42(2.33,2.51)
		22.5-27.5	Crude	5014	178332	1.42(1.38,1.46)
		27.5-32.5	Crude	12865	649747	1
		32.5-37.5	Crude	3560	202020	0.89(0.85,0.92)
		37.5-42.5	Crude	772	41042	0.95(0.89,1.02)
		≥42.5	Crude	76	3367	1.14(0.91,1.43)
	Maternal	<22.5	Adjusted	NA		1.07(1.01,1.14)
		22.5-27.5	Adjusted			1.02(0.98,1.07)
		27.5-32.5	Adjusted			1
		32.5-37.5	Adjusted			1.07(1.02,1.12)
		37.5-42.5	Adjusted			1.31(1.22,1.41)
		≥42.5	Adjusted			1.63(1.31,2.04)
	Paternal	<22.5	Crude	1328	27154	2.47(2.35,2.61)
		22.5-27.5	Crude	3488	120658	1.46(1.41,1.51)
		27.5-32.5	Crude	14844	749697	1
		32.5-37.5	Crude	3472	199265	0.88(0.85,0.91)
		37.5-42.5	Crude	1366	75813	0.91(0.87,0.96)
		≥42.5	Crude	809	42122	0.97(0.90,1.04)
	Paternal	<22.5	Adjusted	NA		2.36(2.23,2.49)
		22.5-27.5	Adjusted			1.44(1.39,1.49)
		27.5-32.5	Adjusted			1
		32.5-37.5	Adjusted			0.86(0.83,0.88)
		37.5-42.5	Adjusted			0.86(0.82,0.90)
		≥42.5	Adjusted			0.88(0.82,0.94)
Kim et al, 2020	Maternal	≤20	Crude	50	302	2.63(1.94,3.57)
		21-25	Crude	504	5925	1.34 (1.20,1.49)
		26-30	Crude	1011	16017	1
		31-35	Crude	415	5530	1.19(1.06,1.33)
		36-40	Crude	115	1049	1.73(1.42,2.10)
		≥41	Crude	17	135	1.95(1.18,3.21)

	Maternal	≤20	Adjusted	42	276	2.19(1.57,3.05)
		21-25	Adjusted	491	5588	1.27(1.13,1.41)
		26-30	Adjusted	1049	15159	1
		31-35	Adjusted	430	5183	1.20(1.07,1.36)
		36-40	Adjusted	106	956	1.60(1.30,1.98)
		≥41	Adjusted	15	119	1.82 (1.05,3.17)
	Paternal	≤20	Crude	18	97	2.89(1.74,4.79)
		21-25	Crude	151	1543	1.51(1.27,1.79)
		26-30	Crude	805	11904	1.04(0.94,1.14)
		31-35	Crude	772	11843	1
		36-40	Crude	251	2994	1.29(1.12,1.48)
		≥41	Crude	60	556	1.65(1.27,2.15)
	Paternal	≤20	Adjusted	15	89	2.36(1.36,4.08)
		21-25	Adjusted	115	1145	1.38(1.15,1.66)
		26-30	Adjusted	805	11285	0.98(0.88,1.08)
		31-35	Adjusted	815	11203	1
		36-40	Adjusted	244	2772	1.21(1.05,1.40)
		≥41	Adjusted	45	502	1.22(0.90,1.65)
Sauver et al, 2004	Maternal	＜20	Crude	19	373	0.96(0.59,1.56)
		20-30	Crude	227	4225	1
		＞30	Crude	59	1103	0.99(0.73,1.34)
	Maternal	＜20	Adjusted	19	373	0.80(0.47,1.35)
		20-30	Adjusted	227	4225	1
		＞30	Adjusted	59	1103	1.11(0.80,1.53)
	Paternal	＜20	Crude	5	117	1.22(0.68,2.13)
		20-30	Crude	179	3473	1
		＞30	Crude	101	1819	1.07(0.82,1.32)
	Paternal	＜20	Adjusted	5	117	0.58(0.21,1.61)
		20-30	Adjusted	179	3473	1
		＞30	Adjusted	101	1819	1.16(0.88,1.52)

Sciberras et al, 2011	Maternal	≤18	Crude	NA		2.05(0.93,4.51)
		19-37	Crude			1
		⩾38	Crude			0.93(0.68,1.23)
Silva et al, 2014	Maternal	＜20	Crude	885	2239	1.63(1.48,1.78)
		20-24	Crude	3005	9177	1.21(1.15,1.28)
		25-29	Crude	4433	15465	1
		30-34	Crude	3265	11543	0.98(0.93,1.04)
		35-39	Crude	1231	4033	1.09(1.01,1.18)
		40+	Crude	172	605	0.99(0.83,1.18）
Wang et al,2019	Maternal	＜20	Adjusted	755	14839	1.29(1.19,1.40)
		≥20	Adjusted	15714	322902	1
	Paternal	＜20	Adjusted	390	7802	1.27(1.13,1.42)
		≥20	Adjusted	16079	329939	1
	Maternal	＜20	Crude	700	14393	1.14(1.04,1.24)
		≥20	Crude	15171	317119	1
	Paternal	＜20	Crude	353	308791	1.20(1.09,1.32)
		≥20	Crude	15518	22721	1

Table S2. Quality assessment of the included studies (case-control studies)

Study	Selection				Comparability	Exposure			Overall quality assessment score (of a maximum of 9)
	Is the case definition adequate?	Representativeness of the cases	Selection of controls	Definition of Controls	Comparability of cases and controls on the basis of the design or analysis	Ascertainment of exposure	Same method of ascertainment for cases and controls	Non-Response rate	
Chudal R et al, 2015	*Yes, with independent validation	* consecutive or obviously representative series of cases	* community controls	*No history of disease	*Study controls for maternal smoking during pregnancy, number of previous births	* Secure record	* Yes	Non respondents described	8
Gustafsson et al, 2011	*yes, with independent validation	* consecutive or obviously representative series of cases	* community controls	*No history of disease	*Study controls for maternal smoking, Year of birth, weeks of gestation, Apgar score	* Secure record	* Yes	non respondents described	8
Kim et al, 2020	yes, eg record linkage or based on self reports	potential for selection biases or not stated	* community controls	*No history of disease	*Study controls for child's sex, age, parental educational level, current exposure to secondhand smoking	written self report or medical record only	* Yes	Non respondents described	6
Sauver et al.	yes, eg record	* consecutive or obviously	* community controls	*No history of disease	*Study controls for	* Secure record	* Yes	*Same rate for both	8

2004	linkage or based on self reports	representative series of cases			child's sex, low birth weight, birth to a single parent, sex, education level,			groups	
Silva et al, 2014	*yes, with independent validation	* consecutive or obviously representative series of cases	* community controls	*No history of disease	*Study controls for moking in pregnancy, preterm labor, oxytocin use， sex	* Secure record	* Yes	*Same rate for both groups	9

TableS3. Quality assessment of the included studies (cohort studies)

Study	Selection				Comparability	Outcome			Overall quality assessment score (of a maximum of 9)
	Representativeness of the exposed cohort	Selection of the non exposed cohort	Ascertainment of exposure	Demonstration that outcome of interest was not present at start of study	Comparability of cohorts on the basis of the design or analysis	Assessment of outcome	Was follow-up long enough for outcomes to occur	Adequacy of follow up of cohorts	
Chang et al. 2014	* Truly representative of the average population in the community	* Drawn from the same community as the exposed cohort	* Secure record	No	** Study controls for offspring's sex, birth order ， birth year ， paternal age at childbirth	* Record linkage	* yes	*complete follow up - all subjects accounted for	8
Galera et al, 2011	* Truly representative of the average population in the community	* Drawn from the same community as the exposed cohort	* structured interview	*Yes	** sex, premature birth, Prenatal tobacco exposure, Low birth weight, Prenatal alcohol exposure, Prenatal illegal drug exposure	self report	* yes	No statement	7
Hvolgaard et al, 2017	* Truly representative of the average population in the community	* Drawn from the same community as the exposed cohort	* Secure record	* Yes	** Study controls for Birth date, maternal smoking during pregnancy, gender, and gestational age	* Record linkage	* yes	*Subjects lost to follow up unlikely to introduce bias (94% follow up)	9
Janecka	* Truly	* Drawn	* Secure record	* Yes	** Study	*	* yes	No statement	8

et al, 2019	representative of the average population in the community	from the same community as the exposed cohort			controls for parity, sex, and parental psychiatric history	Record linkage			
Sciberras et al, 2011	* Truly representative of the average population in the community	* Drawn from the same community as the exposed cohort	* structured interview	*Yes	*Maternal cigarette smoking, maternal postnatal depression, male gender	self report	* yes	*Subjects lost to follow up unlikely to introduce bias (89% follow up)	7
Wang et al,2019	* Truly representative of the average population in the community	* Drawn from the same community as the exposed cohort	* Secure record	*Yes	* Study controls for birth year, income, maternal education, race history of comorbidity, history of psychiatric disorders, prepregnancy obesity	* Record linkage	* yes	*Subjects lost to follow up unlikely to introduce bias (96% follow up)	8

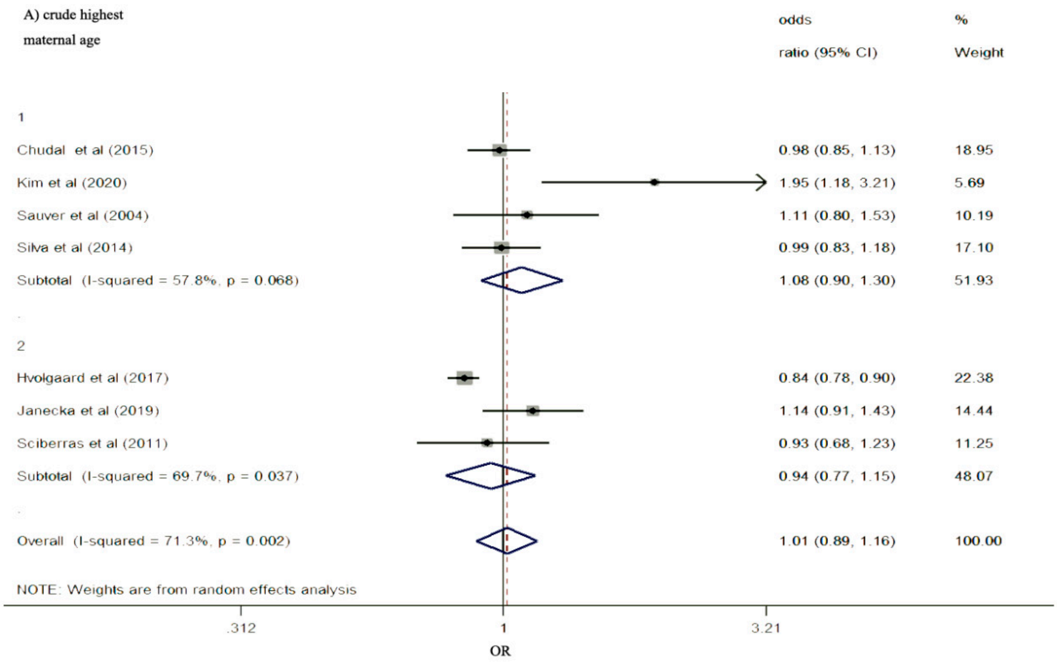

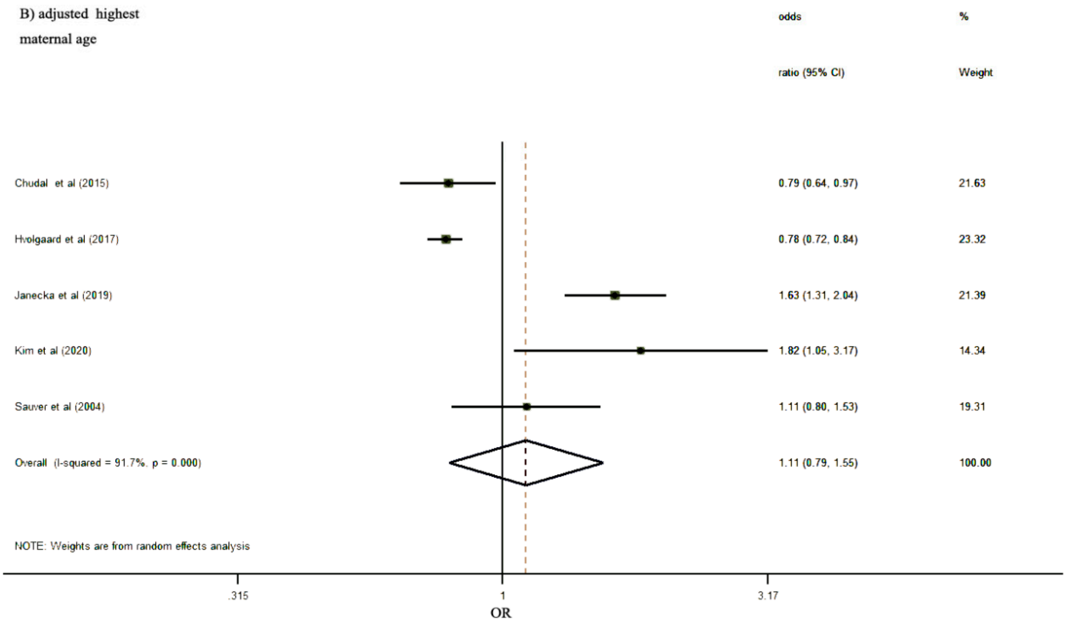

Fig. S1. Risk of ADHD according to the highest maternal age category vs. reference points. Pooled crude effects (A) and adjusted effects (B) for maternal age from random-effects meta-analyses are shown

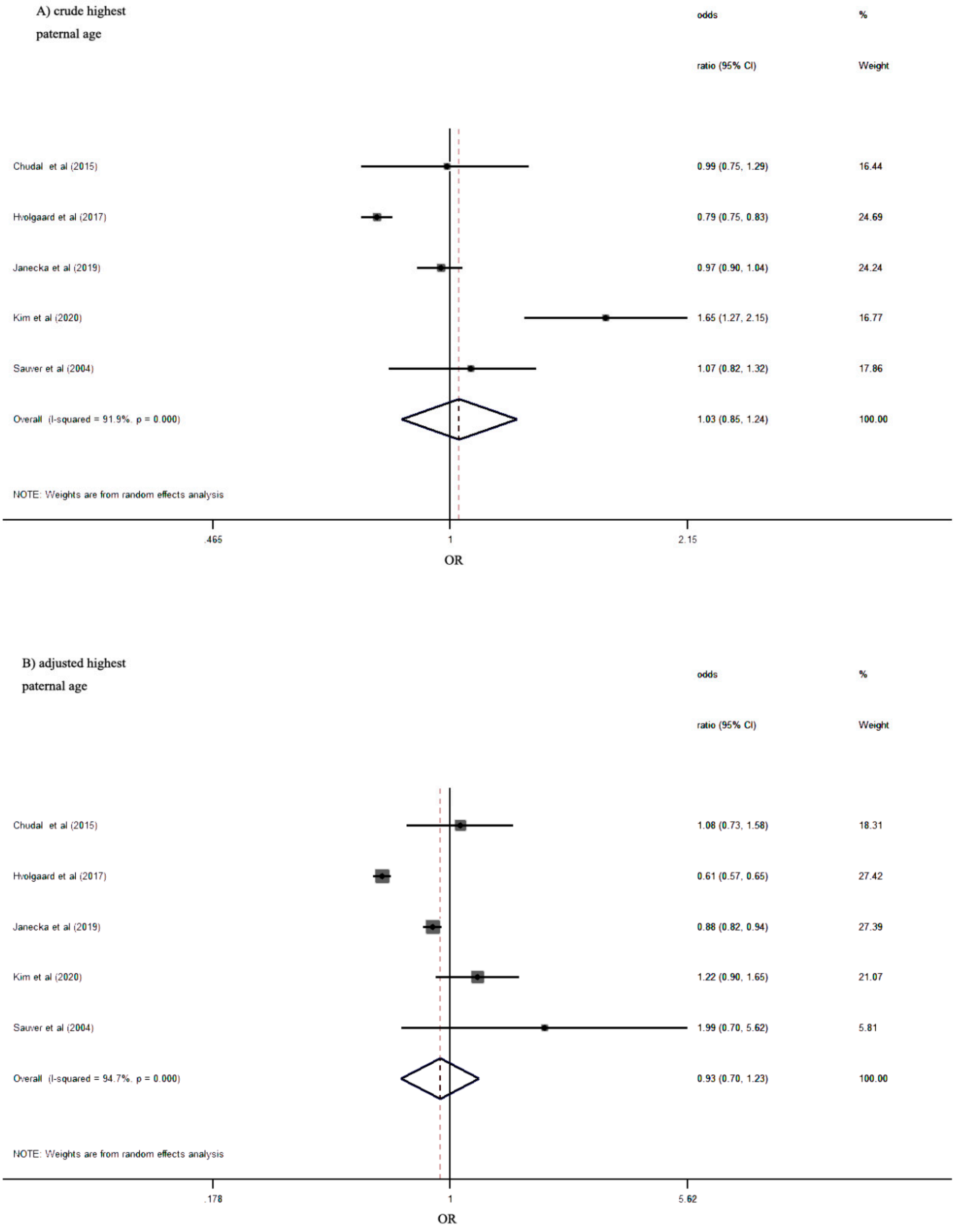

Fig. S2. Risk of ADHD according to the highest paternal age category vs. reference points. Pooled crude effects (A) and adjusted effects (B) for paternal age from random-effects meta-analyses are shown
